# Sepsis-induced immunosuppression: mechanisms, diagnosis and current treatment options

**DOI:** 10.1186/s40779-022-00422-y

**Published:** 2022-10-09

**Authors:** Di Liu, Si-Yuan Huang, Jian-Hui Sun, Hua-Cai Zhang, Qing-Li Cai, Chu Gao, Li Li, Ju Cao, Fang Xu, Yong Zhou, Cha-Xiang Guan, Sheng-Wei Jin, Jin Deng, Xiang-Ming Fang, Jian-Xin Jiang, Ling Zeng

**Affiliations:** 1 Department of Trauma Medical Center, Daping Hospital, State Key Laboratory of Trauma, Burns and Combined Injury, Army Medical University, Chongqing, 400042 China; 2 Department of Respiratory Disease, Daping Hospital, Army Medical University, Chongqing, 400042 China; 3 Department of Laboratory Medicine, the First Affiliated Hospital of Chongqing Medical University, Chongqing, 400016 China; 4 Department of Critical Care Medicine, the First Affiliated Hospital of Chongqing Medical University, 400016 Chongqing, China; 5 Department of Physiology, School of Basic Medicine Science, Central South University, Changsha, 410078 China; 6 Department of Anesthesia and Critical Care, the Second Affiliated Hospital and Yuying Children’s Hospital of Wenzhou Medical University, Zhejiang 325027 Wenzhou, China; 7 Department of Emergency, the Affiliated Hospital of Guizhou Medical University, Guizhou Medical University, 550001 Guiyang, China; 8 Department of Anesthesiology, the First Affiliated Hospital, Zhejiang University School of Medicine, Hangzhou, 310052 China

**Keywords:** Sepsis, Immunosuppression, Immune monitoring, Immunomodulatory therapy

## Abstract

Sepsis is a common complication of combat injuries and trauma, and is defined as a life-threatening organ dysfunction caused by a dysregulated host response to infection. It is also one of the significant causes of death and increased health care costs in modern intensive care units. The use of antibiotics, fluid resuscitation, and organ support therapy have limited prognostic impact in patients with sepsis. Although its pathophysiology remains elusive, immunosuppression is now recognized as one of the major causes of septic death. Sepsis-induced immunosuppression is resulted from disruption of immune homeostasis. It is characterized by the release of anti-inflammatory cytokines, abnormal death of immune effector cells, hyperproliferation of immune suppressor cells, and expression of immune checkpoints. By targeting immunosuppression, especially with immune checkpoint inhibitors, preclinical studies have demonstrated the reversal of immunocyte dysfunctions and established host resistance. Here, we comprehensively discuss recent findings on the mechanisms, regulation and biomarkers of sepsis-induced immunosuppression and highlight their implications for developing effective strategies to treat patients with septic shock.

## Background

Sepsis is a common posttraumatic complication and one of the major causes of long-term mortality in combat casualties. Currently, it is defined as a life-threatening organ dysfunction syndrome caused by dysregulated host response to infection [1], which is attracting increasing attention in military and civilian medicine. Mortality of sepsis has gradually decreased with the timely use of antibiotics, fluid resuscitation, and multiple organ support therapies over the past couple of decades. There is still significant mortality and room for improvement. A recent Global Burden of Diseases report showed that in 2017, a total of 48.9 million cases of sepsis were reported worldwide, with a mortality rate of 22.5%, accounting for nearly 20% of all global deaths [2–4]. In addition to the high health-related burden, septic shock is one of the most expensive pathological conditions to treat, with an estimated annual health care burden of $24 billion [5]. Patients with sepsis have different disease stages, although recent studies found that both phases are dynamic and take turns or exist simultaneously. Septic patients may die due to immunosuppression or due to reactivation of primary infection-induced excessive inflammation early or late. The occurrence of immunosuppression is increasingly recognized as a critical factor in sepsis mortality, especially after discharge. Therefore, understanding the pathological role of sepsis-induced immunosuppression is crucial for disease prevention and treatment [6, 7].

**Fig. 1:**
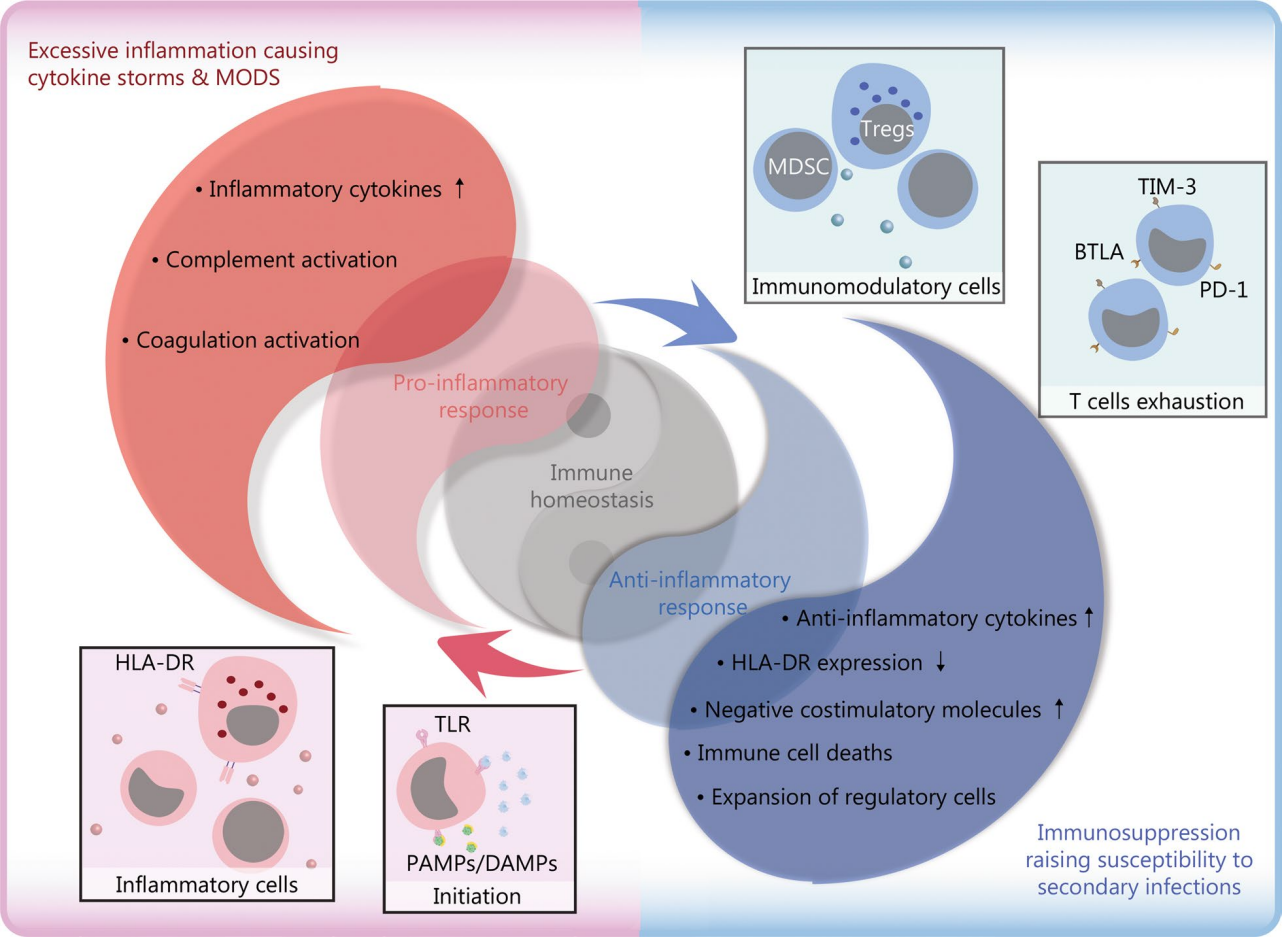
Schematic diagram of immune homeostasis imbalance in sepsis. The immune response is initiated when the host recognizes PAMPs and DAMPs. Inflammatory cells release pro-inflammatory cytokines and cause excessive inflammation in the early stage of inflammation. Under physiological conditions, the dynamic balance between pro-inflammatory and anti-inflammatory responses maintains immune homeostasis. However, after the onset of sepsis, the balance is disrupted. The upregulated expression of pro-inflammatory cytokines released by inflammatory cells and the activation of the complement and coagulation systems, result in excessive inflammation, which further leads to cytokine storms and MODS. Concurrently or subsequently, the increased release of anti-inflammatory cytokines and coinhibitory molecules, decreased expression of HLA-DR, death of immunocytes, and expansion of regulatory cells lead to immunosuppression, increasing the susceptibility to secondary infections, which is the main cause of poor prognosis in septic patients. TLR toll-like receptor, PAMP pathogen-associated molecular pattern, DAMP damage-associated molecular pattern, HLA-DR human leukocyte antigen-DR, MODS multiple organ dysfunction syndromes, Treg regulatory T cell, TIM-3 T-cell immunoglobulin domain and mucin domain-3, BTLA B and T lymphocyte antigens, PD-1 programmed cell death 1

During sepsis, inflammation and immunosuppression may occur sequentially or concurrently. During the early stage of the systemic inflammatory response, if the immune system promptly clears pathogens, the immune balance can be quickly restored. If the pathogens were not removed in time, they will result in an imbalance of immune regulation. In that case, patients are prone to secondary infections, leading to long-term immunosuppression, immune collapse, and even physical disabilities, also known as persistent inflammation immune-suppression catabolism syndrome, which is also common in other critical illness arising from other sterile insults, such as subsequent major trauma, pancreatitis and cardiopulmonary bypass [8]. Specifically, sepsis survivors had a mortality rate of 15% in the first year after discharge and 6–8% in the next 5 years [9, 10]. As shown in Fig. 1, immune homeostasis plays a crucial role in sepsis pathophysiology and determines clinical outcomes.

The two major categories of the immune system are the innate immune system and the adaptive immune system, which can release many inflammatory cytokines early in sepsis to eliminate foreign pathogens. Specifically, innate immune cells act as the first line of defense to recognize pathogens or pathogen-associated molecular patterns (PAMPs). The complement system is also activated concurrently as a prominent feature of the pro-inflammatory response with the release of C3a and C5a. This can result in the release of damage-associated molecular patterns (DAMPs), such as high mobility group box 1 (HMGB1), generated by dead or dying cells, which further promotes innate immune cell activation and cytokine release, such as tumor necrosis factor-alpha (TNF-α) and interleukin 1 beta (IL-1β) [11]. However, many components of innate immunity have paradoxical effects on immune homeostasis regulation. An uncontrolled inflammatory response promotes the activation of the coagulation system and the formation of intravascular microthrombosis, leading to disseminated intravascular coagulation (DIC) [12, 13]. Worse still, excessive release of pro-inflammatory molecules may lead to organ failure, tissue damage and immunodeficiency by regulating T-cell apoptosis [14, 15]. Another study further confirmed that C5a could suppress the antimicrobial functions of neutrophils by disrupting phagosomal maturation and cause immune defects in critical illness [16].

Excessive inflammatory responses and cytokine storms during sepsis have long been considered major causes of high mortality. However, drugs targeting TNF-α, IL-1β, or toll-like receptors have not achieved satisfactory clinical results in improving the survival rate of patients with sepsis [17, 18]. Recent preclinical and clinical studies have shown that innate immune dysfunction and suppressed acquired immunity simultaneously drive multiple organ damage and septic death [19, 20]. Therefore, maintaining the inflammatory and anti-inflammatory balance and the normal functioning of innate and acquired immune functions are equally important [6].

Sepsis-induced immunosuppression is derived from disorders of innate and acquired immunity. It is characterized by the release of anti-inflammatory cytokines, death of immunocytes, T-cell exhaustion, and excessive production of immunomodulatory cells, including regulatory T cells (Tregs) and myeloid-derived suppressor cells (MDSCs). Decreased expression of human leukocyte antigen-DR (HLA-DR) and increased expression of immune checkpoint molecules [such as programmed cell death 1 (PD-1), T-cell immunoglobulin and mucin domain-containing protein-3 (TIM-3), and B and T lymphocyte attenuator (BTLA)] further aggravate immunosuppression. Consequently, inflammation-related immunosuppression is a crucial factor leading to secondary infection and multiple organ dysfunction syndrome (MODS), which is the main cause of poor prognosis in septic patients [20, 21].

In this review, we summarize the current research progress on the mechanism of sepsis-induced immunosuppression from the following aspects: increased release of anti-inflammatory cytokines, immunocyte death, decreased HLA-DR expression, increased expression of negative costimulatory molecules, and expansion of immunomodulatory cells. We also introduce sepsis immune status monitoring and immunomodulatory drug treatment, which may help clinicians better understand sepsis-induced immunosuppression.

## Mechanism of sepsis-induced immunosuppression

Long-term sepsis leads to immunosuppression, characterized by a large amount of immune cell dysfunction and the activation of multiple signaling pathways. Below, we focus on well-known mechanisms of sepsis-induced immunosuppression.

### Increased release of anti-inflammatory cytokines

**Fig. 2:**
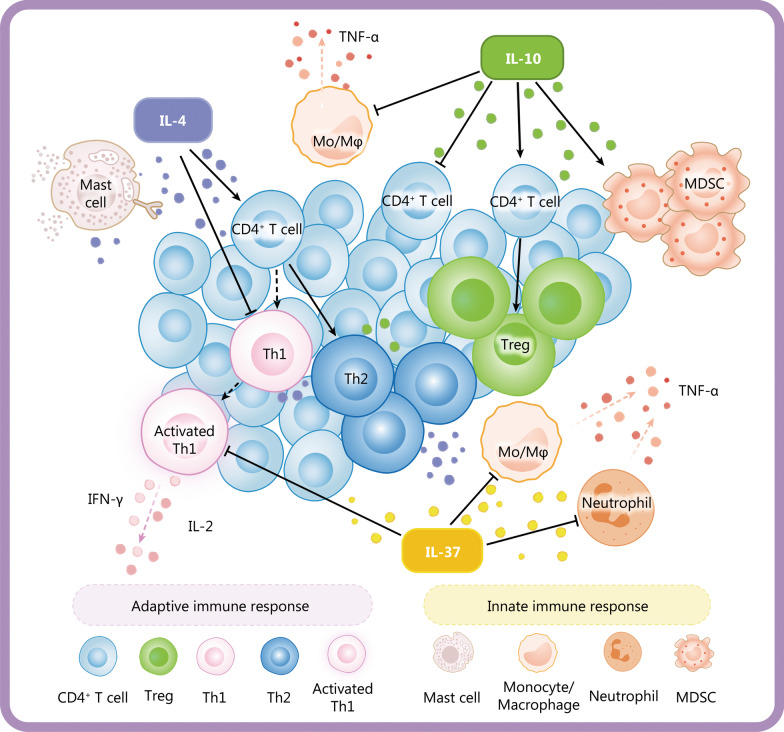
Anti-inflammatory cytokines in sepsis. Anti-inflammatory cytokines mainly include IL-4, IL-10 and IL-37. IL-4 can induce CD4^+^ T cells to differentiate into Th2 cells and promote autocrine signaling of mast cells through positive feedback. It can also stimulate the release of other anti-inflammatory cytokines and inhibit the release of pro-inflammatory cytokines such as IL-2 and IFN-γ by activated Th1. IL-10 may aggravate immunosuppression by decreasing the release of pro-inflammatory cytokines including TNF-α, inhibiting the proliferation of CD4^+^ T cells and promoting the differentiation of CD4^+^ T cells into Tregs and the proliferation of MDSCs. IL-37 is closely related to the severity of sepsis-induced immunosuppression by suppressing the pro-inflammatory cytokine release from monocytes and neutrophils. Inflammatory cytokines such as TNF-α, IFN-γ and IL-2 are represented by red dots whereas IL-10, IL-37 and IL-4 are represented by dots in other colors. Th1 T helper 1, Th2 T helper 2, MDSC myeloid-derived suppressor cell, TNF-α tumor necrosis factor-alpha, IFN-γ interferon-γ, IL interleukin, Mo/Mφ monocyte/macrophage

Sepsis-related anti-inflammatory cytokines mainly include IL-4, IL-10, and IL-37 (Fig. 2). IL-4 is produced and secreted by activated T cells and mast cells. Its biological characteristics mainly include: 1) inducing the differentiation of CD4^+^ T cells into T helper 2 (Th2) cells; 2) promoting autocrine signaling through positive feedback to produce other anti-inflammatory cytokines; and 3) inhibiting the release of pro-inflammatory cytokines [21]. In sepsis, the release of IL-4 is increased, leading to the differentiation of naive T cells into Th2 cells, and the maturation of Th2 cells can be blocked by anti-IL-4 antibodies [22, 23]. In addition, IL-4 and IL-10 decrease the release of pro-inflammatory cytokines, including IL-2 and interferon-γ (IFN-γ), by inhibiting the differentiation of CD4^+^ T cells toward Th1 cells. Anti-IL-4 monoclonal antibodies can reverse this process.

IL-10 is mainly secreted by monocytes/macrophages and Th2 cells. It is an immunosuppressive cytokine with multiple functions: 1) inhibiting T-cell proliferation and function; 2) inhibiting the release of pro-inflammatory cytokines; and 3) promoting the proliferation of immunosuppressive cells, such as Tregs and MDSCs [24]. IL-10 inhibits the expression of TNF-α in monocytes of septic mice. In contrast, the administration of anti-IL-10 antibody promotes the release of TNF-α from monocytes and the release of IFN-γ from Th1 cells [25]. IL-10 can also promote the proliferation of MDSCs in mice with sepsis and aggravate immunosuppression in mice with advanced sepsis [26, 27]. Similar functions of IL-10 in suppressing T-cell proliferation, promoting Treg production, and limiting effector cytokine production are observed in patients with sepsis [28].

Unlike pro-inflammatory members of the IL-1 family (e.g., IL-1β), IL-37 generally reduces innate inflammation and adaptive immune responses. It is produced by immunocytes and can inhibit pro-inflammatory cytokine release and antigen presentation [29]. The expression of IL-37 in patients with sepsis is significantly upregulated, which could hinder the proliferation and release of pro-inflammatory cytokines and is closely related to the severity of sepsis-induced immunosuppression [30]. Another study also demonstrated that IL-37 could significantly downregulate the expression of HLA-DR and CD86 in septic mice and inhibit antigen presentation, indicating that IL-37 has an immunosuppressive effect in sepsis [31].

### Loss of immune effector cells

#### Immunocyte apoptosis

**Fig. 3:**
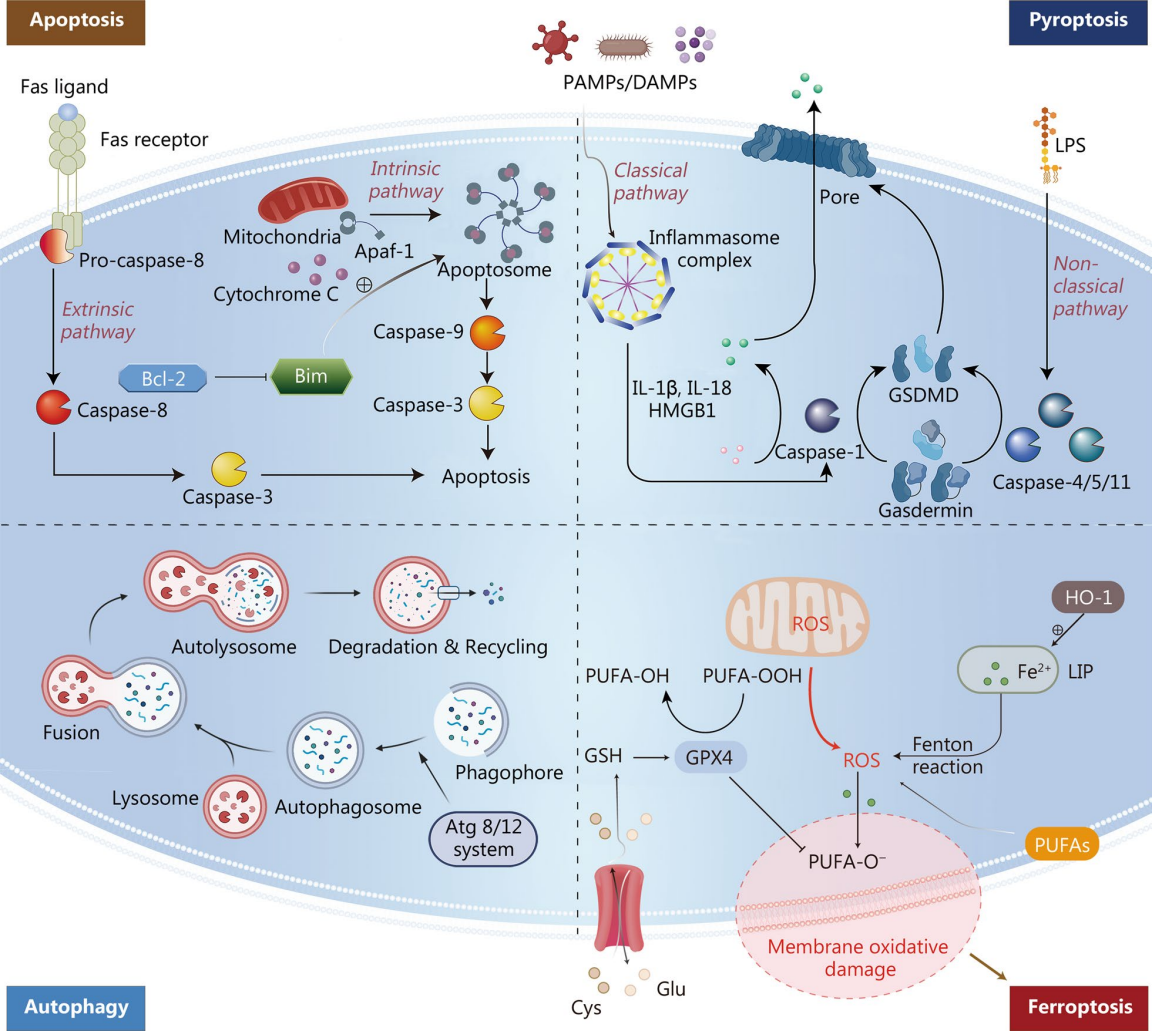
Four types of cell death in sepsis. Apoptosis: Apoptosis is activated in either the extrinsic or the intrinsic pathway. The extrinsic pathway is triggered by the Fas/FasL pathway after infection. The death receptor Fas activates caspase-8 by binding to the Fas ligand that is expressed on activated T lymphocytes during cellular immunity followed by activation of caspase-3 to trigger the execution pathway of apoptosis. In the intrinsic pathway, death stimuli including DNA damage and the accumulation of misfolded proteins break the balance between proapoptotic and antiapoptotic signals mediating mitochondrial outer membrane permeabilization after which cytochrome C is released from mitochondria and forms an apoptosome with Apaf-1. The apoptosome activates caspase-9 and finally activates caspase-3. The pro-apoptotic protein Bim accelerates apoptosis while the anti-apoptotic protein Bcl-2 inhibits apoptosis. Pyroptosis: In the classical pathway, the inflammasome complex activates caspase-1 upon simulating PAMPs and DAMPs. Caspase-1 promotes the release of pro-inflammatory cytokines such as IL-1β IL-18 and HMGB1 and then cleaves gasdermin into GSDMD. GSDMD aggregates into a pore on the cell membrane. In the non-classical pathway, LPS activates caspase-4, caspase-5 and caspase-11 which cleave gasdermin into GSDMD to form the pore and drive pyroptosis. Autophagy: Atg 8/12 systems activate the phagophore to form the autophagosome. The autophagosome fuses with the lysosome and further form an autolysosome. Lysosomal enzymes degrade misfolded proteins and damaged organelles in autolysosomes and enter the recycling process. Ferroptosis: Ferroptosis is a ROS-dependent form of cell death defined by iron-dependent accumulation and lipid peroxides that is resulted from an imbalance between the synthesis of oxidants and antioxidants. At the core process, PUFAs and lipids containing PUFAs are particularly sensitive to oxidation by enzymes and nonenzymatic processes such as iron-dependent Fenton reactions to form lipid hydroperoxides that can produce toxic lipid free radicals (e.g., alkoxyl radicals) in the presence of iron. Furthermore, by taking protons from neighboring PUFAs, these free radicals might initiate a new round of lipid oxidation and spread oxidative damage. GPX4 functions as a phospholipid hydroperoxidase in the redox system to reduce phospholipid hydroperoxide production and plays an anti-ferroptosis role. The enzyme HO-1 can accelerate the formation of a labile iron pool and further promote lipid peroxidation. GSDMD gasdermin-D, LPS lipopolysaccharide, Atg autophagy-related gene, ROS reactive oxygen species, PUFA polyunsaturated fatty acid, LIP labile iron pool, GPX4 glutathione peroxidase 4, HMGB1 high mobility group box 1, IL interleukin. Part of the autophagy was created partially utilizing the templates on BioRender.com as a reference

Apoptosis is a form of regulated cell death that aims to remove damaged cells and maintain homeostasis under physiological conditions [32]. Apoptotic pathways include extrinsic and intrinsic pathways (Fig. 3). In the extrinsic pathway, caspase-8 is activated by the Fas/Fas-ligand pathway, which subsequently activates caspase-3 to trigger the execution of the apoptotic program. In the intrinsic pathway, cytochrome C is released from mitochondria to the cytoplasm to form apoptosomes with apoptotic protease activating factor-1 (Apaf-1). The apoptosome activates caspase-9 and finally activates caspase-3. The mitochondrial apoptosis pathway is regulated by members of the B-cell lymphoma/leukemia-2 (Bcl-2) family. For example, the proapoptotic protein Bim exacerbates apoptosis, while the antiapoptotic protein Bcl-2 inhibits apoptosis [31, 33]. The expression levels of cytochrome C, Bim, caspase-3, caspase-8, and caspase-9 are significantly increased in a cecal ligation and puncture (CLP) mouse model, while the expression of Bcl-2 is inhibited, thereby promoting T-cell apoptosis [34]. IL-33 prevents T lymphocyte apoptosis and improves the survival rate of the CLP model by reducing the expression of Fas and upregulating the expression of Bcl-2 [35]. Apoptosis and Fas expression in peripheral blood mononuclear cells of children with sepsis are significantly increased and positively correlated [36]. These findings suggest that inhibiting apoptosis may be a strategy to restore immune function to defend against infection.

#### Immunocyte pyroptosis

Pyroptosis is a form of inflammatory cell death primarily mediated by caspase-dependent gasdermin (GSDM) family activation. Pyroptosis is characterized by the loss of membrane integrity, cell swelling and rupture, followed by the release of pro-inflammatory intracellular contents [37, 38]. Immunocyte pyroptosis during sepsis mainly exerts biological effects through classical and non-classical pathways (Fig. 3). The classical pathway activates caspase-1 through the inflammasome. Activated caspase-1 promotes the release of pro-inflammatory cytokines, such as IL-1β, IL-18, and HMGB1, recruiting immune cells to aggregate the inflammatory response. Activated caspase-1 recognizes and cleaves gasdermin-D (GSDMD), and cleaved GSDMD form a pore in the cell membrane, resulting in pyroptosis. In the non-classical pathway, cytosolic lipopolysaccharide (LPS) binds to caspase-4/5 in humans or caspase-11 in mice, activating GSDMD and further leading to pyroptosis [39–41]. The cleavage and activity of GSDMD are fine-tuned by lipid peroxidation inhibited by glutathione peroxidase 4 [40].

Compared with that in healthy patients, the expression of caspase-1 in multiple immune cells is increased in septic patients. The release of IL-18 and the percentage of monocyte pyroptosis are correlated with the occurrence of sepsis [42]. In addition to caspase-1, increased expression and activity of caspase-11 in macrophages are observed in LPS-treated septic mice. Knockout of caspase-1 reduces septic mortality in mice [43]. The activity of bone marrow-derived macrophages in septic mice treated with HMGB1 is inhibited, resulting in increased release of IL-1β and IL-18. As expected, the knockout of caspase-11 or GSDMD in macrophages inhibited IL-1β and IL-18 release [44]. HMGB1 released by hepatocytes is crucial in caspase-11-dependent pyroptosis and lethality in LPS-mediated endotoxemia and bacterial sepsis. Blocking hepatocyte HMGB1 prevents pyroptosis in sepsis [45]. Myeloid STING (also known as TMEM173) mediates GSDMD-related immunocoagulation by releasing tissue factor III (F3) in bacterial sepsis independent of the IFN response [46]. The inhibition of the TMEM173-GSDMD-F3 pathway blocks DIC and increases animal survival of sepsis [46]. Since anaplastic lymphoma kinase (ALK) activates STING during sepsis [47], it is expected that clinically used ALK inhibitors (e.g., ceritinib) might block GSDMD activation in macrophages. Nevertheless, inhibiting STING-dependent immune mediator (such as sequestosome 1) release and the induction of cell death may provide different approaches to block inflammation-related immunosuppression during sepsis [48, 49].

#### Other types of cell death

In addition to apoptosis and pyrosis, autophagy and ferroptosis contribute to sepsis-induced immunosuppression. The dual effect of autophagy during sepsis leads to growing interest. Due to its homeostasis-maintaining properties, studies have shown that autophagy protects the immune system by clearing pathogens, stabilizing the mitochondrial membrane, and preventing apoptosis of immunocytes [50–53]. However, inhibiting autophagy enhances the antibacterial ability of macrophages [54, 55]. Although autophagy primes neutrophils and contributes to the formation of neutrophil extracellular traps [56, 57], it also attenuates inflammation and mediates tolerance to *Staphylococcus aureus* α toxin [58]. In addition, blocking autophagy ameliorates cytokine storms and vascular leakage [59]. These results indicate that autophagy might regulate the release of inflammatory mediators and lead to a relatively immune-tolerant status.

Ferroptosis, a reactive oxygen species-α-dependent form of cell death defined by iron-dependent accumulation and lipid peroxides that differs from other types of cell death in biochemistry, morphology, and primary regulatory mechanisms [60], plays a role in the onset and progression of sepsis [61, 62]. Recent research has demonstrated that ferroptotic cells release DAMPs and activate downstream signaling pathways, thus aggravating organ failure caused by sepsis [63]. Identifying ferroptosis-specific DAMPs may help assess the effects of ferroptosis on immune cell function. Glutathione peroxidase 4 (GPX4) functions as a phospholipid hydroperoxidase in the redox system to reduce phospholipid hydroperoxide production and plays an anti-ferroptosis role [64]. T-cell-specific Gpx4-deficient mice are more sensitive to viral and parasitic infections due to T-cell death caused by ferroptosis [65]. Senescent erythrocytes release heme, hemin, and hemoglobin, which cause ferroptosis in platelets, monocytes, and macrophages and induce immunosuppression by negatively regulating the STAT1 pathway [66]. In addition, labile heme can be catalyzed by the enzyme heme oxygenase-1 (HO-1), whose expression and activity increase in the late phase of sepsis. Additionally, HO-1 can promote the shift from Th1 to Th2 and induce immune cell death, contributing to immunosuppression in sepsis [67]. These investigations have all shown that ferroptosis is closely related to sepsis development and septic shock. Ferroptosis inhibitors might provide new perspectives on clinical sepsis treatment.

### Expansion of regulatory cells

The maintenance of immune system homeostasis relies on the dynamic balance of pro-inflammatory and anti-inflammatory factors. In addition to dysfunction or loss of effector cells, overactivation of regulatory cells, including Tregs and MDSCs, also plays a crucial role in sepsis-induced immunosuppression.

#### Tregs

Upon first being described in the 1960s, Tregs have been well studied because of their indispensable roles in sepsis [68]. Accounting for 5% to 10% of CD4^+^ T cells, Tregs play a vital role in maintaining immunological homeostasis and self-tolerance. In sepsis-induced immunosuppression, the frequency of Tregs in peripheral blood increases, which is associated with long-term mortality in septic patients [69]. The mechanisms of Treg-induced immunosuppression have not been well elucidated. To date, the main mechanisms include: 1) releasing anti-inflammatory cytokines, including TGF-β and IL-10 [70–73]; 2) upregulating the negative costimulatory receptors on immune effector cells, including TIM-3, PD-1, T cell Ig and ITIM domain (TIGIT), cytotoxic T lymphocyte antigen-4 (CTLA-4) and neuropilin-1 [74–77]; 3) epigenetic modifications of the *Foxp3* gene, which enhance the stability of Tregs during lymphopenia [78, 79]; and 4) metabolic shift of Tregs from glycolysis to oxidative phosphorylation that enhances their suppressive capacity [80, 81]. These results suggest that decoding the role of Tregs in sepsis-induced immunosuppression might provide a potential target for future research.

#### MDSCs

MDSCs are a diverse group of immature myeloid cells, including the progenitors of monocytes, neutrophils, and dendritic cells (DCs) that suppress innate and adaptive immune responses [82]. Granulocytic/neutrophilic MDSCs and monocytic MDSCs are the two main subsets of MDSCs [83]. Following stimulation by inflammation, both subsets are released from bone marrow and migrate to lymph nodes to inhibit the proliferation of lymphocytes in infected mice [84]. In accordance with this, sepsis causes a shift of normal neutrophils to suppressor cells in the bone marrow, which culminates with an enormous increase in MDSCs [85]. The migration and aggregation of MDSCs in the liver and spleen during sepsis were also observed [86]. Chronic immune suppression in septic patients was also reported to be associated with increased MDSCs, which was also correlated with the incidence of nosocomial infections after the diagnosis of sepsis [87]. In patients with sepsis, the number of immature myeloid cells increased at least 6 weeks after infection. Only MDSCs obtained at, and beyond 2 weeks post-sepsis can significantly inhibit T lymphocyte proliferation and IL-2 production. This may be the cause of chronic and persistent immunosuppression in patients with sepsis [88]. However, the phenotype of MDSCs still lacks consistency [89]. Therefore, since MDSCs may be therapeutic target cells for sepsis-induced immunosuppression, it is necessary to study their phenotypes further.

### Decreased expression of HLA-DR

HLA-DR is a major histocompatibility complex class II molecule expressed on monocytes, macrophages, and other immune cells, and it is critical for activating the adaptive immune system. These cells present pathogen proteins and express a protein complex co-binding with T-cell receptors (TCRs). High expression levels of HLA-DR correspond to well-activated immune function in mice, thus supporting HLA-DR as an indicator for assessing immune status in patients with sepsis. However, the mechanism of reprogramming the decrease of HLA-DR is still uncertain. It has been reported that reduced HLA-DR correlates with reduced cytokine responses to LPS [90]. For septic patients, decreased expression of HLA-DR in bone marrow monocytes is closely related to the clinical prognosis of sepsis [91]. The expression of HLA-DR in patients with sepsis is 70% lower than that in nonseptic patients. HLA-DR expression is inversely correlated with sequential organ failure assessment (SOFA) scores [92]. A frequency of HLA-DR-positive monocytes less than 30% is an indicator of immunosuppression [93]. These findings suggest that HLA-DR is a biomarker for immunosuppression and adverse clinical outcomes in patients with sepsis [94].

### Increased expression of negative costimulatory molecules

Negative costimulatory molecules (also known as immune checkpoints), such as PD-1, programmed cell death ligand 1 (PD-L1), TIM-3, CTLA-4, BTLA, lymphocyte activation-gene-3 (LAG-3) and 2B4, are expressed by different immune and nonimmune cells. Functionally, they inhibit innate immune cell function (phagocytosis, pathogen clearance, and cytokine release) and lead to T-cell exhaustion, a state of T-cell dysfunction that occurs during inflammatory stimulations and cancers. In preclinical models of sepsis, inhibitors and antibodies designed to block the engagement of negative costimulatory molecules with immunocytes have been shown to improve immune cell functions and increase host resistance to sepsis [95]. The interaction between PD-1 on the surface of T cells and PD-L1 on the surface of antigen-presenting cells (e.g., DCs) leads to T-cell exhaustion, which is mainly manifested by weakened effector T-cell function, reduced cytokine production, and inhibited cell proliferation [5]. Increased expression of PD-1 indicates a poor clinical prognosis in septic patients [96, 97]. The expression of PD-1 and PD-L1 in neutrophils and monocytes in patients with septic shock is significantly higher than that in uninfected patients in the intensive care unit (ICU) and positively correlated with sepsis severity and mortality [98].

Immune checkpoint inhibitors have achieved great success in the treatment of certain cancer patients. Hotchkiss et al. [99] conducted the first clinical safety and pharmacokinetic assessments of the anti-PD-1 antibody nivolumab in septic patients. The administration of nivolumab does not lead to safety concerns, which strongly supports further initiation of the clinical therapeutic application of testing anti-PD-1 antibodies in sepsis [99]. In addition to the PD-1 and PD-L1 axes, TIM-3 expression is significantly increased on CD4^+^ T cells in patients with sepsis. Conditional knockout of TIM-3 in CD4^+^ T cells protects infectiously immunosuppressed mice from death by reducing organ dysfunction [100]. CTLA-4 transmits inhibitory signals to T cells and inhibits their activation by binding to CD80 and CD86. In the CLP mouse model, the expression of CTLA-4 is upregulated on CD4^+^ and CD8^+^ T cells. CTLA-4 antibody treatment attenuates sepsis-induced apoptosis and improves survival in a CLP sepsis model [101]. The expression of CTLA-4 on CD4^+^ T cells in patients with sepsis is significantly increased, suggesting that CTLA-4 is a potential target for sepsis treatment [96]. The significance of the expression profiles of negative costimulatory molecules at different stages of sepsis requires further evaluation, which may shed light on new targets for therapeutic interventions.

In conclusion, sepsis-induced immunosuppression is caused by immune cell death and dysfunction after the upregulation of negative costimulatory molecules and excessive release of cytokines by immune cells. A comprehensive understanding of the pathogenesis may provide new ideas and strategies for treating sepsis-induced immunosuppression.

## Immune status monitoring of sepsis

The pathological process of sepsis-induced immunosuppression results from disturbances and dysfunctions of the innate and adaptive immune systems. Mechanisms of the innate immune system dysregulation include dysfunctional neutrophil recruitment and migration, aberrant macrophage differentiation and regulation, suppression of DC immune function, impaired natural killer (NK) cytotoxicity and cytokine production, and overactivation of the complement system. Dysfunction and decreased T cells, increased Treg frequency, imbalanced Th17/Treg ratio, impaired B-cell function, and low immunoglobulin concentrations are hallmarks of acquired immune system dysfunction. An observational retrospective study showed that monitoring lymphocyte counts, monocyte counts, and the neutrophil-to-lymphocyte ratio (NLR) could predict the severity and 28-day mortality of septic patients with intra-abdominal infections [102]. Monitoring septic immune status is crucial for assessing the prognosis and timely protection of organ function in patients with sepsis.

### Innate immune function monitoring

#### Neutrophil function monitoring

Neutrophils capture and kill pathogens through various antimicrobial activities. The chemiluminescence intensity of neutrophils in septic patients is significantly reduced. A reduction in their bactericidal activity is associated with the severity of sepsis-induced immunosuppression, especially in patients with poor prognoses [103]. Activation of some neutrophil proteins may serve as potential biomarkers for a sepsis diagnosis, such as CD64 and human triggering receptors expressed on myeloid cells-1 (TREM-1). The expression of CD64 on neutrophils is low under physiological conditions but increases significantly after stimulation by pro-inflammatory cytokines during bacterial infection [104]. TREM-1 consists of membrane TREM-1 (mTREM-1) and soluble TREM-1 (sTREM-1). sTREM-1 in serum and urine is more sensitive for the early diagnosis of sepsis than C-reactive protein (CRP) and procalcitonin (PCT). Hence, this assay may prove to be a valuable method for identifying patients at risk of infection and monitoring the immune status of these septic patients. CD88 is another marker for neutrophil dysfunction mediated by C5a. Excessive release of C5a inhibited the expression of CD88 and caused neutrophil defects in phagocytosis by inhibiting RhoA activation and polymerization [105]. Reduced expression of CD88 on neutrophils was strongly associated with increased subsequent secondary infection and was a strong predictor of immunosuppression [106].

#### Monocyte/macrophage antigen presentation

HLA-DR expression is a robust marker of the antigen presentation ability of monocytes. In many clinical trials, monocyte HLA-DR (mHLA-DR) has been used as an indicator of innate immunity. mHLA-DR in septic patients is significantly reduced compared to healthy controls [107, 108]. Approximately 3–4 d after the initiation of septic shock, mHLA-DR in the septic shock death group was significantly reduced compared with that in the survival group. Therefore, mHLA-DR is a representative indicator of the immune status of septic patients. A prospective observational study found the HLA-DR level was an independent predictor of the prognosis of septic patients. In contrast, nearly half of the patients experienced alterations in immune status from day 1 to day 3 after the onset of sepsis [109]. Meanwhile, as a substudy of the REAnimation Low Immune Status Marker (REALISM) program [110], assessing the REAnimation Low Immune Status Marker Test (REALIST) score might be a novel tool to stratify patients at risk of secondary infections. The REALIST score measures the mHLA-DR, the percentage of CD10^−^CD16^−^ immature neutrophils and the serum IL-10 level of patients from day 5 to day 7. It can predict 30-day mortality [111]. Continuous observation of mHLA-DR can evaluate the immune status and predict the outcome of septic patients [112, 113]. Low mHLA-DR expression levels are typical in discharged patients and patients with poor prognoses. During the 6-month follow-up period, most patients recovered to near-normal levels. The expression of mHLA-DR is highly consistent with the CD4^+^ T-cell count, confirming that mHLA-DR is a reliable immune status indicator [114]. mHLA-DR is also an important monitoring indicator of immune status in septic patients during immunoregulation therapy and provides critical information when evaluated as a dynamic variable over time [115].

#### Cytokine release by monocytes/macrophages

TNF-α is a pro-inflammatory cytokine mainly produced by monocytes/macrophages. During the stage of sepsis-induced immunosuppression, the release of TNF-α by monocytes is significantly reduced. TNF-α secretion is limited in LPS-stimulated monocytes isolated from the peripheral blood of sepsis-induced immunosuppressive patients compared with nonseptic patients. Therefore, in septic patients, LPS-stimulated TNF-α secretion by monocytes at a serum concentration below 200 ng/L can be used as a cutoff to diagnose sepsis-induced immunosuppression [116]. The ability of monocytes to produce TNF-α is enhanced after immunostimulant therapy in patients with sepsis. In vitro LPS-stimulated monocytes also release less IL-12 during sepsis-induced immunosuppression, whereas peripheral blood monocytes from the surviving sepsis group secrete more IL-12 [117]. Study has shown that IL-10 monitoring from day 1 to day 3 after the onset of sepsis may help predict the prognosis of septic patients [109]. According to Peronnet et al. [118], monitoring CD74 and IL-10 on day 1 and day 3 after admission could predict the occurrence of nosocomial infections. Septic patients with less IL-10 released by peripheral blood monocytes have better prognoses [119]. Regardless of the source, they are an important part of the cytokine storm, producing different immune functions depending on the concentration.

#### NK-cell function monitoring

NK cells are the main effector cells in innate immunity and can recognize and attack viruses and bacteria. They play an important role in the pathophysiology of sepsis [120]. NK-cell activity is used to monitor the immune status of tumor chemotherapy patients but is rarely reported in sepsis. The NK-cell count decreases significantly in patients with sepsis, while patients with a high proportion of NK cells/lymphocytes have a better prognosis [121]. NK cells exert cytotoxic effects by producing a variety of cytokines, the most representative of which is IFN-γ. The serum concentration of IFN-γ reflects the function of NK cells [122]. Damaged effector functions of NK cells might be a critical mechanism of immunosuppression during sepsis. After stimulation by phorbol-12 myristate 13-acetate (PMA) and ionomycin, the production of IFN-γ and TNF-α by CD3^−^CD56^+^ NK cells in septic patients is impaired [123]. Longitudinal studies examining changes in the proportions of different NK subtypes during infection in septic patients may help to optimize treatment and identify biomarkers that can help predict disease severity.

### Adaptive immune function monitoring

Patients with persistently low lymphocyte counts for 3–4 d may be immunosuppressed [124–126]. Although lymphocyte count lacks good specificity, it is the most widely used test in clinical practice. A persistent lymphocyte count below 1.0 × 10^9^/L indicates abnormal immune status. Patients with persistently low lymphocyte counts have higher mortality rates and a higher risk of developing chronic infections [127].

#### T-cell count

The T-cell count is also an indicator for predicting sepsis-induced immunosuppression [128]. During the process of immunosuppression, due to increased apoptosis and high expression of inhibitory immune checkpoint molecules, the number of T cells is significantly reduced, further aggravating immunosuppression and even leading to immune collapse [129, 130]. The CD4^+^ T-cell count and CD8^+^ T-cell count can be detected by flow cytometry. The changes in T-cell subsets in septic patients also have clinical predictive value. For example, a decrease in the CD4^+^/CD8^+^ T-cell ratio indicates acquired immune abnormalities [131]. A decrease in the CD4^+^/CD8^+^ T-cell ratio in trauma patients is directly related to the risk of sepsis and is correlated with the occurrence of MODS [132]. However, the clinical significance of T-cell subset monitoring is less stable than mHLA-DR. There is no significant difference in the proportion of CD4^+^/CD8^+^ T-cells between septic patients and nonseptic patients [115]. Thus, comparing the distribution of different subtypes of T cells in blood and tissues is essential for a dynamic understanding of sepsis-related adaptive immunity.

#### T-cell proliferation and secretion function

Infection affects the adaptive immune response characterized by decreased T-cell proliferation, increased apoptosis, and abnormal cytokine secretion. The lymphocyte count in the peripheral blood of patients with sepsis is reduced, and most of the viable lymphocytes are in an unresponsive state. The ability of T cells to proliferate in patients with severe trauma is significantly decreased, which is closely related to the severity of the injury and the high mortality rate of patients with sepsis, suggesting that T-cell proliferation is in a state of persistent low response, causing immune disorders [133]. A study on memory CD4^+^ T-cell proliferation during recovery from septic lymphopenia showed that bone marrow is the primary site of CD4^+^ T-cell homing and proliferation during sepsis-induced immunosuppression [134]. Bone marrow CD4^+^ T cells have a higher baseline proliferation rate than splenic T cells [134]. The monitoring of cytokine secretion is one of the crucial indicators for evaluating the function of T cells. An observational study identified that from day 2 to day 3, the frequency of granzyme B (GrzB)/perforin-positive CD8^+^ T cells and CXCR3^+^CD8^+^ T cells might be monitoring indicators of immune status [135]. Inducing T cells to secrete IL-2 and TNF-α. Department of mitogen (including phytohemagglutinin) treatment can be used in the clinical evaluation of T-cell function.

#### T-cell differentiation

During postinjury sepsis, T-cell dysfunction decreases the proliferation of CD4^+^ T cells and induces a shift toward a Th2-type response with accompanying loss of the Th1-type response [136]. The Th17/Treg ratio was strongly positively correlated with the SOFA score, indicating that the higher the Th17/Treg ratio is, the worse the prognosis of patients with sepsis [137]. Th17 cells mediate immune responses by producing the cytokines IL-17, IL-6, and IL-23. Tregs exert immunosuppressive effects by regulating T-cell apoptosis to avoid excessive activation of inflammatory damage [71]. The proportion of Tregs can be detected by flow cytometry with the markers of CD4^+^CD25^+^Foxp3^+^ or CD4^+^CD25^+^CD127^−^. During the typical process of sepsis, the ratio of Th17/Treg cells increases temporarily and then decreases. An abnormal increase in Tregs is accompanied by an inversion of the Th17/Treg ratio, implying the occurrence of immunosuppression [138]. Patients with septic shock often experience immune collapse, characterized by decreased HLA-DR expression and increased Tregs, especially when culminating in death [139, 140]. Thus, clonal populations of T cells can have very different fates and proliferative capacities during sepsis.

#### B-cell function monitoring

Similar to T cells, septic shock is associated with the exhaustion of B lymphocytes [141]. Functionally, serum IgG, IgA, and IgM concentrations directly reflect B-cell status and activity. Peripheral blood immunoglobulin concentrations in septic patients can be used to assess B-cell immune status. The incidence of IgG-deficient hypoglobulinemia in septic patients is as high as 70%, but IgG deficiency has no apparent relationship with clinical prognosis [142]. A recent randomized controlled trial showed no association between a reduction in initial IgG levels and survival in patients with sepsis. In contrast, patients with higher IgG levels tend to have higher mortality [143]. Therefore, it is proposed that the predictive value of a single antibody component for the prognosis of sepsis is not as good as that of multiple antibody components, and the combined application of serum IgG, IgM, and IgA has a better predictive value [144].

The pathological process of sepsis-induced immunosuppression results from disturbances and dysfunctions of the innate and adaptive immune systems. Immunomodulatory therapy has been a research focus for the treatment of sepsis, and the changing immune status and lack of specific clinical signs have prevented the proper application of the therapy. It was suggested that the precise identification of the patient’s immune status would be the first step to provide appropriate immune therapies. Therefore, monitoring the innate/adaptive immune status is crucial for assessing the prognosis and timely protection of organ function in patients with sepsis.

## Activating immunities to fight sepsis

**Fig. 4:**
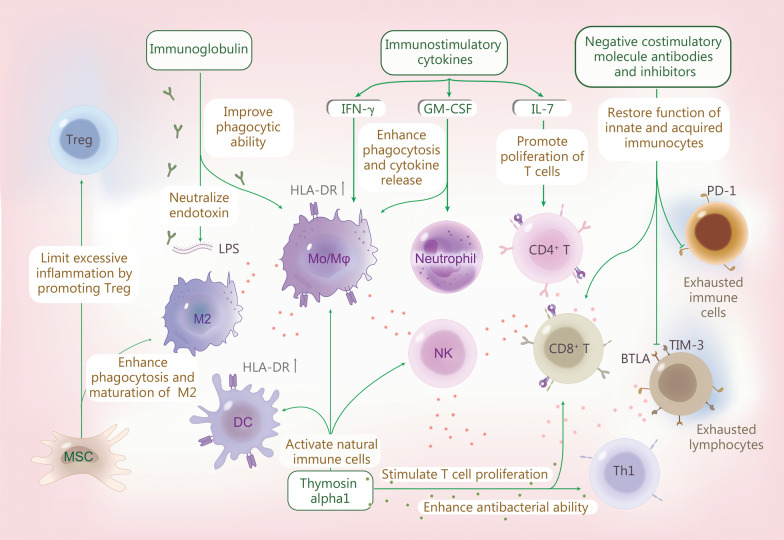
Immunity therapies to fight sepsis. Immunomodulatory therapy includes medication to improve immunity and immune stimulation combined with anti-inflammatory approaches can significantly improve the outcome of severe sepsis. IFN-γ, GM-CSF and IL-7 are immunostimulatory cytokines that have been proven to activate early-responding immune cells in sepsis. IFN-γ and GM-CSF can activate innate immune cells to enhance phagocytosis in addition to pro-inflammatory cytokine release and the expression of mHLA-DR on APCs. IL-7 can increase the number of lymphocytes by promoting proliferation and inhibiting apoptosis. Immunoglobulin is a natural protein that neutralizes endotoxins in the body and promotes the phagocytic ability of monocytes and macrophages. Immunoglobulin therapy may be beneficial in improving the prognosis of septic patients with multidrug-resistant bacterial infections. Thymosin alpha1 can activate innate immune cells such as DCs and NK cells, and macrophages stimulate T-cell proliferation and enhance the antibacterial effect of Th1 cells. MSCs can promote the maturation of M2 macrophages and regulatory T cells, thereby promoting bacterial clearance and limiting excessive inflammation, to alleviate organ damage and ultimately reduce sepsis mortality. Coinhibitory molecule antibodies and antagonists targeting TIM-3, PD-1, BTLA, etc., can restore the function of innate and acquired immunocytes, reversing the immune exhaustion state. GM-CSF granulocyte–macrophage colony-stimulating factor, MSC mesenchymal stem cell, BTLA B and T lymphocyte attenuator, Mo/Mφ monocyte/macrophage, DC dendritic cell, NK natural killer cell, HLA-DR human leukocyte antigen-DR, LPS lipopolysaccharide, PD-1 programmed cell death 1, IFN-γ interferon-γ, IL interleukin

The cause of infectious mortality has long been attributed to an early hyperactivated immune inflammatory response. However, many clinical studies trying to block the excessive inflammatory response found that this did not reduce mortality in sepsis [145]. As shown in Fig. 4, recent studies suggest that activating immunity therapies should be tested as a treatment for refractory sepsis and septic shock.

### Immunostimulatory cytokines

#### IFN-γ

IFN-γ can improve macrophages’ phagocytic and bactericidal ability and enhance their ability to eliminate pathogens. The therapeutic effect of IFN-γ in septic patients has been recently validated. In this study, 9 patients with low expression of mHLA-DR were recruited, and IFN-γ treatment significantly upregulated mHLA-DR expression and increased monocyte secretion of TNF-α, thus helping to eliminate pathogenic bacteria [146]. An ongoing large clinical study (NCT03332225, a verification, and recovery experiment of immune dysfunction in patients with severe infection and sepsis) will further confirm the implication of IFN-γ in treating sepsis [147]. However, because IFN-γ is also a pro-inflammatory cytokine, its clinical safety is of concern.

#### Granulocyte–macrophage colony-stimulating factor (GM-CSF)

GM-CSF can improve immune function by enhancing the phagocytic and bactericidal abilities of neutrophils, monocytes, and macrophages during sepsis. A phase II clinical study of GM-CSF in treating septic patients with respiratory dysfunction showed that intravenous injection of low-dose GM-CSF is beneficial to improve the oxygenation index. Still, it cannot improve the 30-day survival rate [148]. A randomized, double-blind, placebo-controlled clinical study of GM-CSF in septic patients with nontraumatic abdominal infection showed that GM-CSF could shorten the treatment time of antibiotics and reduce infection-related complications, but it failed to reduce the in-hospital mortality rate of septic patients [149]. A randomized, double-blind clinical study observed a significant increase in mHLA-DR expression in all GM-CSF treatment groups compared to only 15.8% in the control group [107]. The ventilator use time, hospital stay, and ICU stay are also significantly shortened in the GM-CSF treatment group [107]. GM-CSF treatment [3 μg/(kg · d) for 4 d] significantly increased neutrophil phagocytosis in 10 septic patients compared with only 44% of neutrophils in the control group. Thus, these findings demonstrate that GM-CSF is beneficial for enhancing the phagocytic capacity of neutrophils, thereby reducing the occurrence of secondary infection [150].

#### IL-7

IL-7 can increase the number of lymphocytes by promoting proliferation and inhibiting apoptosis. A prospective, randomized, double-blind phase II clinical trial on the efficacy of IL-7 in treating sepsis has been completed. A total of 27 septic shock lymphopenia patients were recruited for this study. The results showed that IL-7 treatment does not cause excessive inflammatory reactions or aggravate organ dysfunction but significantly increases CD4^+^ and CD8^+^ T lymphocyte counts in patients with sepsis [128]. Therefore, IL-7 treatment is beneficial for promoting T-cell proliferation in patients with sepsis. However, whether it can alleviate the organ dysfunction caused by sepsis and reduce the mortality rate needs to be further confirmed by phase III clinical studies.

### Immunoglobulin

Immunoglobulin plays a crucial role in neutralizing endotoxin and enhancing the phagocytic ability of monocytes and macrophages. Low immunoglobulin concentrations are seen in most adult sepsis patients [151]. Patients with low immunoglobulin levels and elevated free light chains have impaired immunoglobulin production, which may cause death in septic patients [151]. An interventional cohort study illustrated the potential use of immunoglobulin therapy in multidrug-resistant bacterial infection [152] and sepsis-related coagulation dysfunction [153]. More trials are needed to address the limitations of intravenous immunoglobulin before immunoglobulin therapy can be administered to patients with sepsis, which may provide new ideas and strategies for treating sepsis-induced immunosuppression.

### Thymosin alpha1 (Tα1)

Tα1 is a peptide secreted mainly by the thymus. Tα1 can activate immune cells, such as DCs, NK cells, and macrophages, increase the number of T cells and enhance the antibacterial effect of Th1 cells [154, 155]. Several clinical trials of Tα1 in sepsis have recently been published [115, 156, 157]. A randomized controlled study recruited 42 septic patients showed that patients treated with Tα1 exhibited significantly lower mortality than the control group. Meanwhile, the mechanical ventilation time and ICU stay were shorter in the Tα1 treatment group [156]. Recent studies have shown that Tα1 is also effective in treating COVID-19. A retrospective study recruited 76 severe COVID-19 patients and showed that the mortality of patients treated with Tα1 was significantly reduced compared with that of the control group. Moreover, CD4^+^ and CD8^+^ T-cell counts are increased, whereas PD-1 and TIM-3 expression are downregulated by Tα1 treatment [158]. This study suggested that Tα1 may reduce mortality in severe COVID-19 patients by restoring lymphocyte numbers and reversing T-cell depletion.

### Mesenchymal stem cells (MSCs)

MSCs can alleviate organ damage and ultimately reduce sepsis mortality by ameliorating bacterial clearance, regulating the immune response, reducing apoptosis, and promoting injury repair [159]. The antibacterial effects of MSCs are mediated by the increased release of the antimicrobial peptide LL-37 and increased macrophage endocytosis [160]. Bone marrow mesenchymal stem cells (BMSCs) also play an anti-inflammatory role by inhibiting the activation of the NLRP3 inflammasome/caspase 1 and the release of pro-inflammatory cytokines (such as TNF-α and IL-6). In addition, MSCs promote the maturation of M2 macrophages and Tregs, thereby promoting damage repair and limiting excessive inflammation [161]. Recently, a single-dose clinical trial of MSCs in 9 septic patients showed no safety concerns for using MSCs in septic patients. This study observes no differences in the pro-inflammatory cytokine response between patients injected with MSCs and controls [162].

Two other phase II randomized controlled trials to evaluate the effects of MSCs on the immune response and organ failure in patients with septic shock were launched (NCT03369275 and NCT02883803) [163, 164]. Acute respiratory distress syndrome (ARDS) is a common complication of sepsis and septic shock. MSCs have also been used in the treatment of ARDS. A phase I clinical trial showed that MSCs in the treatment of ARDS appear safe and well tolerated (NTC01902082) [165]. In addition, two clinical trials (NCT02112500 and NCT02444455) are still recruiting patients [166, 167]. These ongoing studies may highlight the potential of MSCs in treating ARDS [168]. However, before MSCs can be successfully applied to the clinical treatment of sepsis, the following issues need to be addressed: (1) What is the effective cell dose of MSCs for sepsis and septic shock therapy? (2) What is the best route of administration? (3) What is the best source of MSCs?

### Negative costimulatory molecule antibodies and inhibitors

Therapies targeting negative costimulatory molecules increase host resistance to infection and improve outcomes. A preclinical study shows that conditional knockout of TIM-3 in CD4^+^ T cells or systemic knockout of TIM-3 reduces immunosuppression-related mortality in a mouse model of sepsis [100]. Another study investigated the ability to block PD-1 to restore innate and acquired immune cell function [169]. BTLA expression causes apoptotic cell loss in primary and secondary lymphoid organs in mice with experimental sepsis. Furthermore, BTLA may be a valuable biomarker for monitoring the development of sepsis. An increased frequency of BTLA^+^CD4^+^ T cells in septic patients is associated with higher rates of subsequent infection [170]. Treatment with anti-PD-1 or PD-L1 antibodies restores neutrophil, monocyte, NK cell, and T-cell function, emphasizing the role of the PD-1/PD-L1 axis in sepsis-induced immunosuppression and the ability of a single immunomodulator to treat infectious diseases [98]. An animal-based study assessed the efficacy of a peptide that inhibits PD-1 and PD-L1 signaling in a second-hit fungal sepsis model. The results show that the peptide significantly improves the survival of septic mice and supports immunotherapy targeting T-cell exhaustion in lethal sepsis [171]. A randomized, double-blind, parallel-group, phase 1b study of 31 septic adult patients diagnosed ≥ 24 h assessed the safety of the anti-PD-1 antibody nivolumab (NCT02960854), suggesting that nivolumab therapy does not result in unexpected safety issues or cytokine storms [99]. Collectively, targeting negative costimulatory molecules has important implications for the treatment of sepsis-induced immunosuppression.

### Immunomodulatory therapy

Hyperactive inflammatory responses and immunosuppression do not occur independently; they frequently coexist in the pathological process of sepsis. Therefore, in recent years, some researchers have proposed a combination of anti-inflammatory and immunomodulatory therapy. Combining anti-inflammatory and immune-enhancing agents can significantly improve the outcome of severe sepsis based on a meta-analysis involving 6 clinical trials [172]. Another meta-analysis of 8 randomized clinical trials confirmed that immunomodulating therapy using ulinastatin (UTI) and Tα1 improves organ function and reduces mortality in patients with severe sepsis [173]. Additionally, a meta-analysis involving 12 clinical studies also showed that severe sepsis therapy with UTI and Tα1 reduces both 28-day and 90-day mortality, whereas treatment with Tα1 alone only reduces 28-day mortality [174]. More high-quality randomized controlled trials are needed to elucidate the role of immunomodulatory therapy in severe sepsis.

In summary, GM-CSF, and T1α treatment may improve the prognosis of septic patients, but the treatment dose needs to be further confirmed. Routine treatment of sepsis with IgG is not recommended except for septic patients with low IgG. For patients with immunosuppression, anti-inflammatory and immune-enhancing treatment can be applied simultaneously. Still, the combination mode, optimal dose, and course of treatment of drugs need to be further confirmed by high-quality clinical studies.

## Future perspectives

Although the understanding of the pathogenesis of sepsis has been dramatically improved in recent years, there is still a long way to go to translate these new findings into effective treatments. This requires a deeper and more comprehensive understanding of the immunopathogenesis of sepsis. New techniques are also being applied to the study of sepsis mechanisms. The high dimension of ‘‘multiomics’’ profiling technology has been used to reveal the complexity of sepsis immunity and inflammation, enabling simultaneous analysis of multiple levels of RNA, proteins, lipids, and metabolites [175]. In addition, combined multiomic analysis could better analyze the systemic responses as well as local tissue-specific responses to sepsis [176].

Machine learning is a subfield of artificial intelligence (AI) that uses data and algorithms to simulate human learning. It can process large amounts of data and detect meaningful patterns of information, thus playing a role in diagnosing and treating sepsis. An AI agent aiming to help physicians use the best dose of fluid and vasopressors for septic patients in the ICU is under development. Based on septic patient characteristics, including demographics, vital signs, and laboratory tests, AI-enhanced learning is used to determine individualized treatment strategies to provide dosages of fluid and vasopressor. The tool helps physicians make decisions but does not replace them. The system is under development for bedside use in a prospective randomized clinical trial in the ICU [177].

Sepsis is a highly heterogeneous syndrome. The heterogeneity of sepsis at the individual patient level hinders the development of the current standard of care for sepsis. This complexity has prompted attempts to develop a precise method for classifying patients into more homogeneous groups with shared biological characteristics, which may aid in developing new individual sepsis therapies. A prospective therapy of individualized sepsis treatment according to subtype (including abdominal, pulmonary, skin/soft tissue, genitourinary and vascular) may provide insights into the outcomes and responses to different sites of infection [178]. Sepsis endotype classification can also aid in providing personalized treatment. A study provides a method for molecularly dividing septic patients into four endotypes at ICU admission. Four sepsis molecular endotypes, Mars1-Mars4, are associated with 28-day in-hospital mortality [179]. Another study used transcriptomic data from adults and children with sepsis to identify three groups of patients as inflammopathic, adaptive, and coagulopathic. The three cohorts had different mortality risks, with the highest in the inflammopathic and coagulopathic groups [180]. In recent years, many important targets for sepsis have been reported, among which *STING* is a vital target gene that mediates the occurrence of sepsis by regulating the production of immune mediators and cell death [181]. Targeting *STING* may also be a new strategy for sepsis-induced immunosuppression [46–48].

The diagnosis and treatment of sepsis have a long way to go, and there is an urgent need to explore new methods of diagnosis and treatment. Currently, clinical studies have preliminarily found that some anti-inflammatory agents and immunotherapies can improve the prognosis of some septic patients [182]. Preliminary clinical studies have found that some anti-inflammatory drugs and immunotherapy can improve the prognosis of some patients with sepsis. However, the key to the success of anti-inflammatory therapy and immunotherapy is to identify the patients who may benefit from specific pathogen infection and immune intervention and to identify the corresponding diagnostic markers for each therapeutic agent. Implementing precision medicine for septic patients, in which immunotherapy is guided by host response biomarkers and reflects targeted pathophysiological changes that drive pathology in a time- and individual-dependent manner will be a significant challenge in the coming years.

## Conclusions

The literature focusing on sepsis-induced immunosuppression was reviewed. The immune response’s status during sepsis results from interactions among multiple mechanisms, including cytokines, cell death, and the expression dynamics of cellular biomarkers. Monitoring the immune status and providing immunomodulatory therapy may improve the survival of patients with sepsis-induced immunosuppression. Understanding the heterogeneity of sepsis, taking the dynamics of different phases of sepsis, and applying precise, individualized therapy are goals of future research.

## Data Availability

Data sharing does not apply to this article, as no datasets were generated or analyzed during the current study.

## Abbreviations

ALK: Anaplastic lymphoma kinase
Apaf-1: Apoptotic protease activating factor-1
ARDS: Acute respiratory distress syndrome
AI: Artificial intelligence
Bcl-2: B-cell lymphoma/leukemia-2
BTLA: B and T lymphocyte attenuator
BMSCs: Bone marrow mesenchymal stem cells
CLP: Cecal ligation and puncture
CTLA-4: Cytotoxic T lymphocyte antigen-4
CRP: C-reactive protein
DAMP: Damage-associated molecular pattern
DCs: Dendritic cells
DIC: Disseminated intravascular coagulation
GM-CSF: Granulocyte–macrophage colony-stimulating factor
GPX4: Glutathione peroxidase 4
GSDM: Gasdermin
GSDMD: Gasdermin-D
HLA-DR: Human leukocyte antigen-DR
HMGB1: High mobility group box 1
HO-1: Heme oxygenase-1
ICU: Intensive care unit
IFN-γ: Interferon-γ
IL: Interleukin
LAG-3: Lymphocyte activation-gene-3
LPS: Lipopolysaccharide
MDSCs: Myeloid-derived suppressor cells
MODS: Multiple organ dysfunction syndrome
MSC: Mesenchymal stem cell
NK: Natural killer cells
PAMP: Pathogen-associated molecular pattern
PCT: Procalcitonin
PD-1: Programmed cell death 1
PD-L1: Programmed cell death ligand 1
PMA: Phorbol-12 myristate 13-acetate
STING: Stimulator of interferon genes
SOFA: Sequential organ failure assessment
Tα1: Thymosin alpha1
TCR: T-cell receptors
Th: T helper cell
TIM-3: T-cell immunoglobulin and mucin domain-containing protein-3
TIGIT: T cell Ig and ITIM domain
TMEM173: Transmembrane protein 173
TNF-α: Tumor necrosis factor-alpha
Tregs: Regulatory T cells
TREM-1: Triggering receptor expressed on myeloid cells-1
UTI: Ulinastatin
